# Medical Opinion and Movement

**Published:** 1910-12-10

**Authors:** 


### Medical Opinion and Movement.

### Deposit of Chlorides in the Skin.

Dr. Wahlgren has already shown that in a normal dog the skin contains the largest quantity of chlorides in the body. Dr. Padtberg, pursuing these researches, is able to arrive at more definite conclusions. He finds that, whether the diet be rich or poor in salt, the skin always contains the greater part of the chlorides and that the muscles hold the smallest amount. With a diet rich in chlorides the skin takes up about a third of the total chlorides. After a diet poor in chlorides this proportion falls to about a quarter. After intravenous injections of hypertonic solutions of sodium chloride the cutaneous tissues are the seat of the greater proportion of the increase in chloride content in the organism. From 28 to 77 per cent. of the chlorides retained in the system are found there. After a diet deprived of chlorides the total chloride content may be diminished by 11 to 21 per cent. From 60 to 90 per cent. of the chlorides eliminated come from the skin, while the other tissues of the body (muscles, blood, intestines) are only subjected to small losses in chlorides. In the dog, therefore, the cutaneous tissues constitute the most important depôt for chlorides in the body. They retain the greater part of the surplus chlorides ingested, and, on the other hand, in case of need, they give up the chlorides the most readily.

### Functions of the Appendix.

Does the appendix serve any useful purpose to the organism? This question has been raised more than once, but has not as yet received any definite reply. It is one of particular interest in view of the recommendations of certain surgeons, notably the Mayos, that the appendix should be removed in cases of laparotomy for other purposes as a preventive measure against possible future trouble. Dr. Härtig, of Grünsfeld, writing on the subject, develops the idea originally put forward many years ago by Lieberkühn that the appendix performs a definite function by reason of its glandular structure. The surface of the appendicular mucous membrane measures from four to six square centimetres, and is able to supply a considerable quantity of mucous secretion, which is poured into the cæcum and helps to "wash out" this somewhat stagnant portion of the digestive tract. In man, in the upright position, the liquid contents of the small intestine are gradually emptied into the cæcum, and are only propelled thence into the ascending colon intermittently when the cæcum is sufficiently distended to cause contraction of its muscular walls. The cæcum is, therefore, always "occupied," and requires a considerable amount of mucous secretion to lubricate and dilute the contents and preserve its mucous membrane from continual irritation. The appendix supplies this extra complement of mucous fluid. It is notable that, apart from man, the appendix is only found in monkeys and in certain kangaroos, that is, in animals assuming a more or less upright position, and in which the cæcum is vertical, or nearly so, and is liable in consequence to stagnation of its contents. In answer to the objection that removal of the appendix does not lead to any untoward result suggestive of any loss to the organism, Dr. Härtig expresses doubts as to the validity of the argument. He recalls a number of patients who still complain after the removal of the appendix, their complaints being generally attributed to adhesions, although in many cases these adhesions are not found to be present, and he thinks that their after-conditions may be attributed to a malfunctioning of the cæcum and a catarrhal typhlitis in consequence of insufficient glandular irrigation.

### Bacterial Toxins.

It is generally considered that bacterial toxins are of an albuminoid nature and belong chemically to that group of substances, although certain observations are not in accord with this theory. Thus Dr. Faust claims to have isolated from cultures of certain micro-organisms a cystalline product of a non-proteid nature, which he terms sepsine. Dr. Burckhardt recently has provided a further example of the possibility of determining more accurately the chemical nature of a bacterial toxin. The *Bacillus putridus* produces a hæmolysin which is very active towards the blood corpuscles of the rabbit. By cultivating this bacillus on bouillon an acid can be extracted with ether, which possesses also strong hæmolytic properties. As a control it was ascertained that fresh bouillon treated in the same way did not yield a hæmolytic etherial extract. Chemical analysis showed that this etherial extract was a non-saturated fatty acid homologous to oleic acid, the hæmolytic properties of which are already established. The acid contains sulphur, and according to the author its constitution corresponds to dimethyl-oxythioleruric acid. The author has not yet succeeded in producing anti-bodies with this acid. Should this be done it would be the first example of the formation of anti-bodies in response to the injection of a definitely known chemical substance.

### Pancreatic Infections.

At a meeting of the Société de Biologie a report was presented on some research work on pancreatic infections carried out by Drs. Abrami, Richet fils, and Saint-Girons. According to these authors most pancreatic inflammatory lesions take place through the blood stream, and not, as usually supposed, by ascending infection up the passage of the ducts from the bowel. Their evidence in support of this view is both clinical and experimental. By systematic examination of the pancreas post-mortem in all manner of diseases they have obtained a considerable amount of evidence of hæmatogenous pancreatic infection. They instance the case of a woman suffering from broncho-pneumonia, in whom they found at the autopsy an abscess at the head of the pancreas with intense canaliculitis and pus in the canal of Wirsung. The pneumo-bacillus was obtained in pure cultivation from these lesions. Similar observations were made in a woman who died from infective jaundice due to the *Bacillus perfringens*, in another pneumonia case, and in two cases of typhoid and in one of erysipelas. The authors have found it quite easy to reproduce the same pathological lesions experimentally in animals by intravenous inoculation, without effecting any traumatic lesion on the pancreas. The frequency of lesions of the canaliculi, which has given rise to the theory of ascending infection up the ducts, is explained by these authors on the ground that the infecting microbes not only settle in the pancreas, but, as in the case of the liver, are eliminated through the excretory ducts. They were able to demonstrate this conclusively. Dogs were submitted to inoculation with certain microbes, and the pancreatic juice collected by means of a canula introduced aseptically into the canal of Wirsung. The juice was immediately cultivated. Under these conditions the typhoid bacillus was recovered in two out of three cases, and the *Bacillus subtilis* in all four cases.

### Bier's Hyperæmia.

For the last year or two there has been a lull in the issue of appreciations or criticisms of Bier's hyperæmic methods of treatment; it seems as if they are passing out of fashion once more in the profession. In the *Edinburgh Medical Journal* Professor MacEwan details some of the conclusions which an extensive trial of venous hyperæmia seem to warrant. He criticises severely some of Bier's statements, but on the whole speaks not unfavourably of the results obtained. The conclusions are thus enunciated: (1) Professor Bier has presented us with a fresh conception of the nature and purpose of inflammation, and has made important additions to our resources for subduing it. (2) In closed tuberculosis venous congestion is limited and uncertain in its effects, and is sometimes productive of harm. In open tuberculosis it is comparatively safe, and yields successful results, especially when the suction method is used. (3) It acts well in circumscribed inflammation, such as carbuncle, abscess, and bursitis, and gives particularly good results in mammary abscess. It is also useful in obstinate septic sinuses; for all these cases suction is the best method. (4) It acts less favourably, and in some cases prejudicially, on spreading inflammation. (5) It gives good results in gonorrhœal arthritis, but should be used with caution in joint suppuration. (6) It is contra-indicated for erysipelas, and is of uncertain value in acute osteomyelitis. (7) It acts beneficially on infected wounds. The majority of these conclusions are in unison with those of Bier and Klapp themselves with the great exception of 2. Those who have tried Bier's treatment in cases of closed tuberculosis according to the rules laid down by Bier and with the special precautions mentioned by him, will hardly agree with Professor MacEwan's opinion.

### Relapses in Malaria.

There exists among men who have dwelt in tropical countries, and therein contracted malaria, an invincible conviction that any and every ailment from which they may suffer after their return is "a touch of fever." No matter what symptoms they have, nor how long it may be since the infection or its latest recurrence, they promptly dose—often indeed overdose—themselves with quinine for days before they think of consulting a doctor. Sir Patrick Manson used to teach that anyone in whom no malarial symptoms had been exhibited for a period of two years on end can consider himself free from the disease, and is only capable of again suffering from it if he acquires a fresh infection. Professor Ross in his recently published book (The Hospital, November 26, 1910, p. 245) notes how scanty the information on this subject of relapses is in the text-books. His reference has evoked from Dr. G. C. Low an account, hitherto unpublished, of a most interesting case which bears directly on this point. In 1900 some infected mosquitoes were sent to England and allowed to bite certain individuals, who, of course, thus got malaria. One of these gentlemen had an attack at the end of September, was given quinine on October 2 of that year, and rapidly recovered. A thorough course of treatment was persevered with, and the patient neither left England nor again submitted himself to experimental inoculation *viâ* the anopheles. In January 1903 he consulted Dr. Low, feeling feverish and out of sorts, and the latter had little difficulty in demonstrating benign tertian parasites in his blood. The lesson to be drawn is that the usual quinine treatment must be prolonged to greater lengths of time than has been usual if relapses are to be abolished entirely. Dr. Low's letter is published in the *Journal of Tropical Medicine and Hygiene*.

### Metal Drains for Central Osteitis.

In a presidential address delivered to the Medical Society of London, and reported in the *Clinical Journal*, Mr. Charters Symonds expounds the indications for the use of metal drainage tubes in certain cases of central osteitis. The cases for which he has used this treatment with success fall into two groups: the chronic central abscess of bone of the type described by Brodie eighty years ago, and the sinus cases with thickening of the bone from chronic infective osteitis and periostitis. In the cases of chronic abscess the lesions are most commonly either tuberculous or the sequelæ of a subsided acute infection. The usual methods of draining such foci of suppuration, after trephining or gouging the thickened bone, are by rubber tubes or gauze packing; but in a considerable proportion the results are not satisfactory, and Mr. Symonds has given up those plans in favour of tubes of aluminium or German silver. The tube is fitted at first with a flange, but this can be dispensed with as soon as the skin has closed round the tube. This it does so effectively that when the tube is removed for cleaning it must be replaced within a few minutes, or it will be impossible to get it in at all. It is not necessary that the tube should be equal in diameter to the cavity in the bone through which it passes. Patients wearing these tubes can carry on their work, and are thus delivered from the chronic state of invalidism into which they otherwise frequently fall. The duration of the drainage required will depend a good deal upon whether it is begun when the abscess is opened or whether after a sinus has existed for some time. The duration is also influenced by the location of the abscess; if it be chronic from the first, and solitary, as in the tuberculous varieties, and the bone is enlarged in an elliptical fashion, reduction soon follows; but acute or subacute sepsis entails a much longer period of drainage. Mr. Symonds first got the suggestion from the late Sir Thomas Smith, but does not know whether the idea originated with him.

### Death from Electric Shock.

In the *Medical Record* Dr. Stanton and Mr. Krida record the observations and experiments on which they base an authoritative exposition of death from electric currents. Low-tension currents tend to kill chiefly by causing cardiac fibrillation. As the tension is increased the effect upon the heart becomes less pronounced, but that upon the central nervous system increases. With high-tension currents death is more likely to be caused by respiratory failure, although if contact be prolonged the heart also is stopped. The evidence points to the nervous system being the chief sufferer from the effects of currents of more than 4,800 volts. Cardiac fibrillation is fatal under known methods of treatment; but the respiratory paralysis of high-tension currents may be successfully treated in certain cases by prolonged artificial respiration. Further investigations should be conducted to see whether the method of Crile and Dolly might not be combined with the high-tension contacts of Leduc or Battellior so as to resuscitate cases of cardiac paralysis seen within five minutes of the accident. Ventricular contractions depend upon a certain pressure in the coronary arteries, as shown by Sollman; a quiescent heart may be made to beat by forcing fluid into these vessels, even though the said fluid be mercury. To bring about this coronary tension, Crile and Dolly first fill the arterial system rapidly by injecting salt solution, through a peripheral artery, towards the heart. When the arteries are nearly filled adrenalin chloride is added to the salt solution. This causes the vessel walls to contract, and rapidly raises the blood pressure. The heart is then stimulated by a few quick compressions of the chest wall, and will often at once begin to beat vigorously. This technique never proved successful in the hands of these authors where once fibrillary contractions had been induced.

### Vaccine Treatment of Diphtheria.

The persistence of the *Bacillus diphtheriæ* in the nose or throat of a patient who is clinically quite recovered from diphtheria is familiar to all; and the importance of such cases in leading to the dissemination of infection is also well recognised. Dr. P. E. W. Smith had such a patient under treatment, and adopted a hint thrown out in Sir A. Wright's "Studies on Immunisation." He publishes details of the case in the *Australasian Medical Gazette*. A girl of eighteen suffered from an uncomplicated attack of diphtheria; the Klebs-Loeffler bacillus was found in practically pure culture, and the usual serum treatment carried out. The membrane speedily disappeared; but a week later a small patch of it recurred and the temperature again rose. A second dose of antitoxin was followed by satisfactory recovery. Swabs were examined every week, but in spite of energetic local treatment they always showed a good growth of the bacilli. At the end of four months the idea of an autogenous vaccine was entertained, and an injection of six million diphtheria bacilli and ten million staphylococci, prepared from the patient's own organisms, was given. This was followed by reaction at the site of injection and by some general disturbance, with a temperature of 100.2. At the end of a week (the throat still containing the specific organisms) another injection was given, the dose being increased to eight million bacilli. There was much less reaction, and a few days later a swab yielded negative results. According to Bosanquet and Eyre, this use of diphtheria vaccine is an established and useful one. But its adoption is as yet far from universal, and increased experience of its value is welcome.

### Syphilitic Reinfection.

The old views as to the extreme rarity of two separate and distinct syphilitic infections have long been proved to be untenable, and many observers have reported cases, which will stand the most careful scrutiny, of undoubted reinfection after the cure of a previous attack. Such an event is really the best proof of the efficiency of the treatment adopted; for the protection afforded by syphilis against again contracting the disease is lost when a complete abolition of the infection has been brought about. In the *Dublin Journal of the Medical Sciences*, Dr. FitzGibbon records an interesting case in which it seems abundantly proved that such a reinfection took place three years and a quarter after the original lesion; this is the shortest period hitherto recorded between two successive infections of this kind. The patient was first seen eight weeks after an admitted exposure to infection; he had then typical chancre, rash, generalised adenitis, mucous patches on the tongue, and snail-track ulceration of the fauces. He was given intra-muscular injections of Lambkin's cream for two and a half months; then a three months' course of potassium iodide; and then a further seven months of injections. Recovery was rapid and complete. Three years and three months later he returned with a fresh chancre and other symptoms of recently acquired generalised syphilis; exposure was admitted eight weeks previously. The same treatment was begun and proved equally satisfactory, though the patient did not persevere with it as long as he was advised to do.

